# Increased cervical disc degeneration in ischemic stroke: a 10-year retrospective data review

**DOI:** 10.1590/1806-9282.20242003

**Published:** 2025-06-16

**Authors:** Aylin Sariyildiz, Ilke Coskun Benlidayi, Ceren Ornek, Burcu Saadet Zeybek, Turgay Demir

**Affiliations:** 1 Cukurova University, Faculty of Medicine, Department of Physical Medicine and Rehabilitation – Adana, Türkiye.; 2 Cukurova University, Faculty of Medicine, Department of Neurology – Adana, Türkiye.

**Keywords:** Cervical spine, Disc degeneration, Disc protrusion, Ischemic stroke

## Abstract

**OBJECTIVE:**

The aim of this study was to evaluate the relationship between cervical spine degeneration and stroke-related parameters in patients with ischemic stroke.

**METHODS:**

The records of the patients with ischemic stroke who had undergone cervical magnetic resonance imaging between October 2013 and 2023 were extracted retrospectively and assessed for eligibility. Age- and sex-matched controls were included for comparative analysis. Disease characteristics (side of stroke, localization, stroke volume, National Institutes of Health Stroke Scale, and affected circulation), presence of cardiac pathology and vascular risk factors, degree of carotid artery stenosis, and inflammatory markers of the patient group were recorded. Computerized measurements and evaluation of some study variables, including Pfirrmann classification, Modic changes, intervertebral disc protrusion, and intervertebral disc height, using magnetic resonance imaging scan were performed for all participants.

**RESULTS:**

A total of 290 patients with ischemic stroke who had undergone cervical magnetic resonance imaging examination were evaluated for eligibility. After applying the exclusion criteria, 45 patients remained (patient group). The median National Institutes of Health Stroke Scale score and stroke volume of the patients were 4 (3) and 1.07 (7.11) mm3, respectively. The Pfirrmann classification also differed between groups (p<0.001). Modic degeneration distribution did not differ between the study groups.

**CONCLUSION:**

The current study confirmed that patients with acute ischemic stroke revealed higher disc degeneration and increased disc protrusion in the cervical spine. Similar underlying mechanisms in stroke and cervical disc degeneration may play a role in these results. This point should be further studied in order to come up with a clear conclusion.

## INTRODUCTION

Stroke is a cerebrovascular disease characterized by the sudden onset of symptoms and clinical signs. Ischemic stroke is the most common type of stroke, accounting for 80% of all cases. It is one of the leading causes of disability and is also a major cause of death worldwide. One in every four cases has recurrent stroke attacks^1–3^. The secondary prevention of stroke starts with the unraveling of possible stroke mechanisms and pathogenesis.

Vascular aging is an important risk factor, especially for atherosclerotic ischemic stroke. Approaches such as controlling hypertension, diabetes, dyslipidemia, and smoking cessation and promoting physical activity, which are among the main targets in stroke reduction, include the control of vascular aging risk factors. Vascular aging is characterized by the gradual development of arterial stiffness due to oxidative stress, endothelial dysfunction, intimal/media thickening, and inflammation, among other pathophysiological features. There is increasing evidence to support that inflammation plays an important role in the pathogenesis of atherosclerosis, endothelial dysfunction, and stroke^4–6^. On the other hand, inflammation has a significant impact on spinal degeneration, which includes the vertebral end-plate, intervertebral discs (IVDs), and facet joints^7–10^. In this regard, it is of value to investigate any potential mechanistic relation between stroke and cervical spine degeneration.

To our knowledge, there are no studies evaluating the relationship between ischemic stroke and cervical disc degeneration in which inflammation plays a fundamental role in their pathogenesis. Therefore, the aim of this study was to evaluate the relationship between cervical spine degeneration and stroke-related parameters in patients with ischemic stroke.

## METHODS

### Study design and recruitment procedures

The current study was conducted at a tertiary university hospital. According to the study design, all data were obtained retrospectively from the hospital database system. The data of patients diagnosed with stroke between October 2013 and 2023 were screened. Those who had undergone cervical magnetic resonance imaging (MRI) examination within a time limit comprising before and after 6 months from the diagnosis were examined for eligibility. The exclusion criteria were: (i) hemorrhagic stroke, (ii) chronic ischemic stroke, (iii) cervical MRI beyond the time limits or of low image quality, (iv) spondylodiscitis, (v) severe cervicothoracic compression fracture, (vi) a history of spinal or cerebral surgery/major trauma or tumor, and (vii) end-stage organ failure. The remaining patients’ demographic data were recorded. Additionally, age- and sex-matched individuals with neck pain and otherwise healthy were included.

The study protocol was approved by the local ethics committee (date: November 3, 2023, number: 138/24). In accordance with the study's retrospective design, informed consent was not required.

### Study parameters

Disease characteristics of the patient group included side of stroke (unilateral, bilateral), localization (cortical, subcortical, brainstem, cerebellar or multiple), stroke volume (mm^3^), National Institutes of Health Stroke Scale (NIHSS) score^11^, affected circulation, presence of cardiac pathology and vascular risk factors, degree of carotid artery stenosis, and inflammatory markers (erythrocyte sedimentation rate [ESR] [mm/h] and C-reactive protein [CRP] [mg/L]).

Computerized measurements and evaluation of some study variables using digitized MRI scan were performed for all participants using Enlil PACS System software version 2.5 (Enlil PACS Viewer, Eroglu Yazılım, Eskisehir, Turkey), as follows:

- **Pfirrmann classification:** The degree of disc degeneration was assessed on T2-weighted midsagittal images with the Pfirrmann classification. According to this classification system, the grading ranges between I and V, with grade V being the highest grade of disc degeneration and characterized by a collapsed disc space^12^.
- **Modic degeneration:** Vertebral end-plate degeneration was assessed in accordance with the classification system proposed by Modic et al.^13^ Modic type I is characterized by a hypointense signal on T1-weighted imaging and a hyperintense signal on T2-weighted imaging, which are indicative of hypervascularity and vertebral body edema. Type II Modic changes exhibit a hyperintense signal on both T1- and T2-weighted imaging, indicative of the fatty replacement of the red bone marrow. Type III Modic changes are characterized by hypointense signals on both T1- and T2-weighted MRI, indicating subchondral bone sclerosis.
- **IVD protrusion:** The protrusion of the IVD was quantified in millimeters based on T2-weighted transverse images. First, a line was drawn at the base of the protruded disc. After this, a perpendicular line was drawn from this line to the most prominent site of the herniated disc^14^.
- **IVD height:** This indicates the average height of the discs as measured in the anterior, middle, and posterior disc zones. IVD height was recorded in millimeters^14^.

### Statistical analysis

Statistical analyses were conducted using the Statistical Package for Social Sciences software (version 22.0; SPSS, Chicago, IL, USA). The normal distribution of data was evaluated by Kolmogorov-Smirnov test. Descriptive statistics were shown as the number of patients (%), mean±standard deviation (SD), and median (interquartile range [IQR]) for categorical, normally distributed continuous, and non-normally distributed continuous variables, respectively. For comparative analyses between patients with ischemic stroke and controls, Student's t-test, Mann-Whitney U test, and Pearson's chi-squared/Fisher's exact test were used for normally distributed continuous data, abnormally distributed continuous data, and categorical data, respectively. p-values were regarded as statistically significant when they were less than 0.05.

## RESULTS

**Figure 1:**
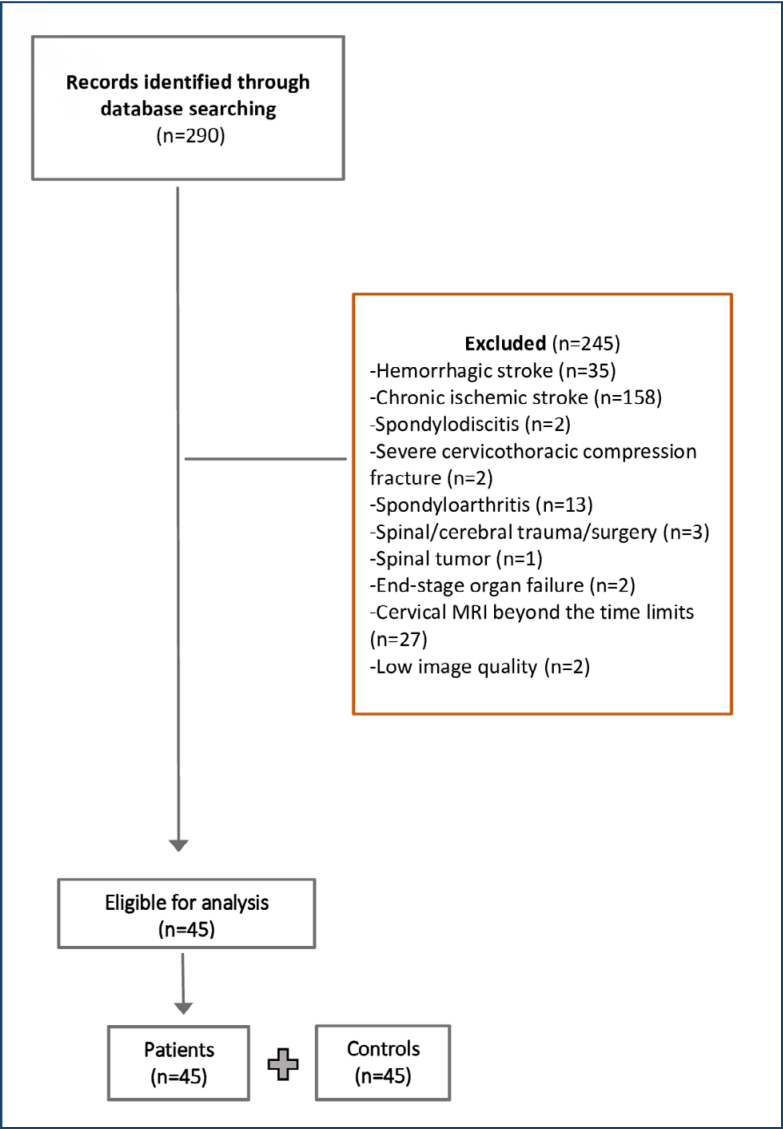
Flowchart of the study.

A total of 290 patients with ischemic stroke who had undergone cervical MRI examination were evaluated for eligibility. After applying the exclusion criteria, 45 patients remained (patient group). A total of 45 age- and sex-matched individuals were included as the control group. The details are presented in the related flowchart (Figure 1).

The mean age of the patient group (32 males and 13 females) was 55.3±10.9 years. Of the patients, 75.6% had unilateral ischemic stroke, while 11 patients had bilateral involvement. In 26.7% of the patients, there was a cardio-embolic etiology. The median NIHSS score and stroke volume of the patients were 4 (3) and 1.07 (7.11) mm^3^, respectively (Table 1).

**Table 1:** Disease characteristics of the patients with ischemic stroke

Variables		Patients (n=45)
Stroke localization (n [%])		
	Cortical	12 (26.7%)
	Subcortical	12 (26.7%)
	Brainstem	5 (11.1%)
	Cerebellar	3 (6.7%)
	Multiple	13 (28.9%)
Side of stroke (n [%])		
	Unilateral	34 (75.6%)
	Bilateral	11 (24.4%)
Etiology		
	Cardioembolic	12 (26.7%)
	Non-cardioembolic	33 (73.3%)
Affected circulation (n [%])		
	Anterior	24 (53.3%)
	Posterior	16 (35.6%)
	Both	5 (11.1%)
	NIHSS	4 (3)
	Stroke volume (mm^3^)	1.07 (7.11)
Cardiac pathology (n [%])		
	Yes	9 (20%)
	No	36 (80%)
Vascular risk factors (n [%])		
	0	15 (34.1%)
	1	12 (27.3%)
	2	10 (22.7%)
	3	7 (15.9%)
Degree of carotid artery stenosis (n [%])		
	No stenosis	26 (57.8%)
	<50%	7 (15.6%)
	>50% or unstable plaque	12 (26.7%)
CRP (mg/L)		6 (6)
ESR (mm/h)		11 (14)

Values are presented as n (%) or median (IQR). NIHSS: National Institutes of Health Stroke Scale; CRP: C-reactive protein; ESR: erythrocyte sedimentation rate.

The details of measurements and comparative data related to cervical MRI parameters are given in Table 2. Accordingly, the patient group revealed increased IVD protrusion when compared to control (2.46±0.84 vs. 1.64±0.84, p<0.001). The Pfirrmann classification also differed between the groups (p<0.001). Almost 80% of the patients had either grade IV or V IVD degeneration, while more than half of the control group revealed grade II or III degeneration at the level of C5–C6. On the other hand, Modic degeneration distribution did not differ between the study groups.

**Table 2:** Comparison of the study variables between patients and controls

Variables		Patients (n=45)	Controls (n=45)	p-value
Age (years)		55.3±10.9	51.1±9.0	0.051
Gender (n [%])				
	Female	13 (28.9%)	13 (28.9%)	>0.999
	Male	32 (71.1%)	32 (71.1%)	
	IVD height (mm)	3.67±1.02	3.82±0.79	0.434
	IVD protrusion	2.46±0.84	1.64±0.84	**<0.001**
Pfirrmann classification				
	Grade I	2 (2.2%)	0	**<0.001**
	Grade II	1 (2.2%)	6 (13.3%)	
	Grade III	8 (17.8%)	22 (48.9%)	
	Grade IV	17 (37.8%)	13 (28.9%)	
	Grade V	18 (40%)	4 (8.9%)	
Modic degeneration				
	None	21 (46.7%)	24 (53.3%)	0.388
	Type 1	4 (8.9%)	1 (2.2%)	
	Type 2	19 (42.2%)	29 (44.4%)	
	Type 3	1 (2.2%)	0	

Values are presented as n (%) or mean±standard deviation. IVD: intervertebral disc. Statistically significant values are denoted in bold.

## DISCUSSION

The current study presented that patients with acute ischemic stroke revealed higher disc degeneration and increased disc protrusion. This finding confirms our hypothesis that patients with ischemic stroke experience increased disc degeneration. IVD degeneration is a common finding on spinal imaging that increases in frequency with age. It is a common cause of spinal pain, which is the leading cause of disability, especially in older adults. The process of IVD degeneration is characterized by gradual structural changes accompanied by severe alterations in metabolic homeostasis^7,15^. Many previous studies have shown that inflammation accompanies disc degeneration and protrusion. The presence of inflammation was thought to be the main difference between symptomatic and asymptomatic IVD degeneration. Studies based on this hypothesis have reported that anti-inflammatory molecules block immune responses and relieve IVD degeneration and pain^7–10,16^. Although the underlying mechanism is similar, there are no studies evaluating the relationship between stroke and cervical disc degeneration.

Age plays a pivotal role in degenerative spinal changes, ischemic cerebral disease, and cardiovascular problems^17^. In order to exclude the impact of age, in the current study, we set a control group with statistically similar ages. Of the patients with ischemic stroke, almost 80% revealed either grade IV or V disc degeneration. On the other hand, less than 40% of the control group revealed such an increased degeneration. Inflammation-related ischemic vascular changes may underlie the increased frequency of degenerative changes in the cervical spine among patients with ischemic stroke. It is already known that cervical arterial pathologies are a causative factor for cerebral stroke^18^. Postmortem angiographic assessments have confirmed that impaired blood flow might play a part in some cervicobrachial disorders, including cervical disc degeneration^19^. The concurrence of cervical disc degeneration and stroke, in the current study, may indicate generalized ischemic vascular changes. Confirming this hypothesis, a recent study showed that spondylosis is a common comorbidity, similar to hypertension, in patients with multiple cerebral infarctions aged between 40 and 93 years^20^.

In terms of vertebral end-plate degeneration, Modic changes did not differ between groups. The prevalence of Modic changes in the lumbar spine is markedly greater than in the cervical spine, leading to a predominance of literature addressing Modic changes in the lumbar region, with fewer studies concerning the cervical spine. Numerous studies have previously reported the close relationship between disc degeneration and vertebral end-plate degeneration in the lumbar spine. Kong et al. evaluated the predictive factors, especially Modic changes, associated with neck pain in patients with cervical disc degenerative disease and reported that severe disc degeneration and loss of disc height were associated with Modic changes in the cervical spine. However, they emphasized that although there is a relationship between disc degeneration and Modic changes in the cervical spine, it cannot be concluded that there is a cause-and-effect relationship between them^21^. Compared to the thoracic and lumbar regions, vertebral end-plate degeneration is less frequent in the cervical region^22^.

The study has several limitations. Since patients’ records were retrieved retrospectively, we were not able to collect some important clinical and sociodemographic data (e.g., neck pain intensity and body mass index). Our study had only a moderate sample size due to the strict inclusion criteria. Considering that the prevalence of Modic changes is less common in the cervical spine, vertebral end-plate changes were not observed in approximately half of the study population (patients and controls), and hence further studies with larger samples are needed. The strengths of the study should also be highlighted. To our knowledge, this is the only study examining the potential relationship between cervical spondylosis and ischemic stroke. Another important strength is examining 10-year hospital data and related cohort.

In conclusion, patients with ischemic stroke have increased cervical disc degeneration signs. The potential causative relation should be thoroughly examined to identify whether patients with severe degenerative changes in the cervical spine are candidates for cerebral stroke. This point should be further studied in order to come up with a clear conclusion.
